# Identification and validation of prognostic signature for breast cancer based on genes potentially involved in autophagy

**DOI:** 10.7717/peerj.9621

**Published:** 2020-07-27

**Authors:** Shanliang Zhong, Huanwen Chen, Sujin Yang, Jifeng Feng, Siying Zhou

**Affiliations:** 1 Center of Clinical Laboratory Science, The Affiliated Cancer Hospital of Nanjing Medical University & Jiangsu Cancer Hospital & Jiangsu Institute of Cancer Research, Nanjing, China; 2 Xinglin laboratory, The First Affiliated Hospital of Xiamen University, Xiamen, China; 3 Department of General Surgery, The First Affiliated Hospital of Nanjing Medical University, Nanjing, China; 4 Department of Medical Oncology, The Affiliated Cancer Hospital of Nanjing Medical University & Jiangsu Cancer Hospital & Jiangsu Institute of Cancer Research, Nanjing, China; 5 The First Clinical Medical College, Nanjing University of Chinese Medicine, Nanjing, China

**Keywords:** Autophagy, Prognosis, Breast cancer

## Abstract

We aimed to identify prognostic signature based on autophagy-related genes (ARGs) for breast cancer patients. The datasets of breast cancer were downloaded from The Cancer Genome Atlas (TCGA) and Gene Expression Omnibus (GEO). Least absolute shrinkage and selection operator (LASSO) Cox regression was conducted to construct multiple-ARG risk signature. In total, 32 ARGs were identified as differentially expressed between tumors and adjacent normal tissues based on TCGA. Six ARGs (IFNG, TP63, PPP1R15A, PTK6, EIF4EBP1 and NKX2-3) with non-zero coefficient were selected from the 32 ARGs using LASSO regression. The 6-ARG signature divided patients into high-and low-risk group. Survival analysis indicated that low-risk group had longer survival time than high-risk group. We further validated the 6-ARG signature using dataset from GEO and found similar results. We analyzed the associations between ARGs and breast cancer survival in TCGA and nine GEO datasets, and obtained 170 ARGs with significant associations. EIF4EBP1, FOS and FAS were the top three ARGs with highest numbers of significant associations. EIF4EBP1 may be a key ARG which had a higher expression level in patients with more malignant molecular subtypes and higher grade breast cancer. In conclusion, our 6-ARG signature was of significance in predicting of overall survival of patients with breast cancer. EIF4EBP1 may be a key ARG associated with breast cancer survival.

## Introduction

It is estimated that 268,600 new cases will be diagnosed with breast cancer in the United States in 2019 and 66,020 will die from this malignancy (Siegel, Miller & Jemal, 2019). Although significant progress has been made in the understanding of cancer biology, identification of risk factors, and new treatments of breast cancer, it is still insufficient in terms of prevention, therapy and prediction of prognosis. Dysregulated gene expression is one of the hallmarks of a variety of diseases including breast cancer (Liu et al., 2019). Expression level of a number of genes has been shown associated with oncogenesis, metastasis, therapy response and prognosis of breast cancer (Liu et al., 2019; Wang et al., 2018; Zhang et al., 2019; Zhou et al., 2019).

Cell death mechanisms in cancer have become the focus of much current research, including apoptosis, necrosis and autophagy (Colen et al., 2015). Autophagy exert distinct functions during the formation and progression of tumor (Romero et al., 2019). Autophagy determines the cells’ fate to die or live, thus facilitating or inhibiting tumorigenesis in context-dependent manner (Romero et al., 2019). Autophagy inhibits tumor growth by preventing the accumulation of reactive oxygen species, organelles and misfolded proteins at the beginning of tumor formation (White, 2015). Depletion of autophagy can lead to an in oxidative stress, DNA damage response and genomic instability (Pajares et al., 2018). On the contrary, high levels of autophagy result in autophagic cell death by deficiency in caspase activation and inappropriate degradation of organelles (Romero et al., 2019).

Autophagy has been shown to participate in development, metastasis, progression and drug resistance of breast cancer (Chen et al., 2019; Tracey et al., 2020; Tyutyunyk-Massey & Gewirtz, 2019; Wang, Yin & Yang, 2019). For example, autophagy was activated as a survival mechanism for breast cancer cells following endocrine therapy or HER2-targeted therapy but failed to rescue the cells (Mele et al., 2020). Estrogen receptor β exerts its inhibitory effect on the migration and invasion of breast cancer cells by inducing the beclin1-dependent autophagic cascade (Song et al., 2019). Potential mechanisms of these effects include providing substrates for basic cell survival and protecting cells from undergoing programed cell death such as apoptosis (Mulcahy Levy & Thorburn, 2020). Pro and anti-apoptotic signals work together to maintain a balance between life and death in normal cells. In cancer cells, autophagy can lead to inefficient mitochondrial outer membrane permeabilization, slowing the rate of cell death and presenting an opportunity for the cell to recover and regain the ability to grow (Mulcahy Levy & Thorburn, 2020). In 2016, Gu et al. (2016) has explored autophagy-related prognostic signature for breast cancer using the datasets from Gene Expression Omnibus (GEO). Recently, Lin et al. (2020) developed a prognostic index based on autophagy-related genes (ARGs) in breast cancer using data from The Cancer Genome Atlas (TCGA). However, the sample size of the first study is relative small, and the second did not validate their prognostic index in other dataset. In present study, we systematically analyzed the expression of ARGs in breast cancer using the data from TCGA and GEO. The Least absolute shrinkage and selection operator (LASSO) was conducted to construct multiple-ARG risk signature for patients with breast cancer (Tibshirani, 1996).

## Materials and Methods

### Data acquisition

A list of ARGs were down from Human Autophagy Database (http://www.autophagy.lu/) (accessed December 2019). All the data used in the present study are publicly accessible from TCGA to GEO. We obtained 1,097 female breast cancer patients with their RNA-Seq data and clinical data from TCGA (accessed December 2019) (Tomczak, Czerwinska & Wiznerowicz, 2015). GEO was searched for the datasets involved with gene expression data of breast cancer; the inclusion criteria include: sample size is no less than 100; raw data of the microarray is available; and clinical outcome of the breast cancer patients is provided. All the data used in the present study are publicly accessible from TCGA (search term: BRCA) and GEO (accession numbers: GSE96058, GSE88770, GSE19615, GSE21653, GSE2990, GES3494, GSE7390, GSE17705, GSE25066, GSE19783).

### Statistical analysis of differentially expressed ARGs

We normalized RNA-Seq data downloaded from TCGA and calculated differentially expressed genes by using DESeq2 (Love, Huber & Anders, 2014) (R package version 1.18.1) in the R software (version 3.4.4). We used Benjamini and Hochberg method (Benjamini et al., 2001) to correct for multiple testing. Differentially expressed genes were screened with the following criteria: fold-change >2 and adjusted *P* value < 0.05 (tumor tissues vs. normal tissues). Then, the differentially expressed ARGs were selected from the differentially expressed genes. We used pheatmap package (R package version 1.0.12) to plot heatmap and ggpubr package (R package version 0.2.4) to draw box plot.

### Enrichment analyses of differentially expressed ARGs

Enrichment analyses including Gene ontology (GO) and Kyoto Encyclopedia of Genes and Genomes (KEGG) were performed using clusterProfiler package (Yu et al., 2012) (R package version 3.6.0). We used Benjamini and Hochberg method (Benjamini et al., 2001) to correct for multiple testing. Adjusted *P* value < 0.05 was considered statistically significant. We constructed a network with cytoscape (version 3.6.0) to illustrate the relationship between genes and enriched GO terms (Shannon et al., 2003). We used GOplot package (R package version 1.0.2) for illustrating the relationship between genes and enriched KEGG pathways.

### LASSO penalized regression analysis

Most useful prognostic markers were selected from ARGs using LASSO Cox regression (Tibshirani, 1996) to construct multiple-ARG risk signature. RNA-Seq data from TCGA were used as training set. Before performing LASSO, we further normalized RNA-Seq data by (log2 (normalized read counts +1)) transformed. LASSO tends to “shrink” the regression coefficients to zero as λ increase. We chose a λ as optimal λ that yielded minimum cross validation error in 10-fold cross validation. We calculated a risk score for all the samples by using the sum of normalized read counts weighted by the coefficients from the LASSO regression (Alencar et al., 2011). Then, we separated the patients into low-and high-risk groups according to the median of the risk score. Finally, overall survival (OS) of the two group patients was evaluated using the log-rank test with survival package (R package version 3.1–7). We used Cox proportional hazards to calculate hazard ratios (HRs) and their 95% confidence intervals (CIs). The “glmnet” package (R package version 2.0–16) was used to do LASSO analysis and a *P* value < 0.05 was considered statistical significance. Receiver operating characteristic (ROC) curve was drawn and the corresponding area under the ROC curve (AUC) was calculated to evaluate the prognostic value of the risk score by using ROCR package (R package version 1.0–7).

### Analyses of GEO dataset

For the datasets generated by the HG-U133A or HG-U133 Plus 2.0 platform, normalization was performed using Robust Multichip Average (RMA) method with affy package (R package version 1.56.0). Regarding the dataset generated by Agilent-014850 Whole Human Genome Microarray, background was corrected using “normexp” method and normalization was conducted using “quantile” method with limma package (R package version 3.34.9).

## Results

### Differentially expressed ARGs

**Figure 1:**
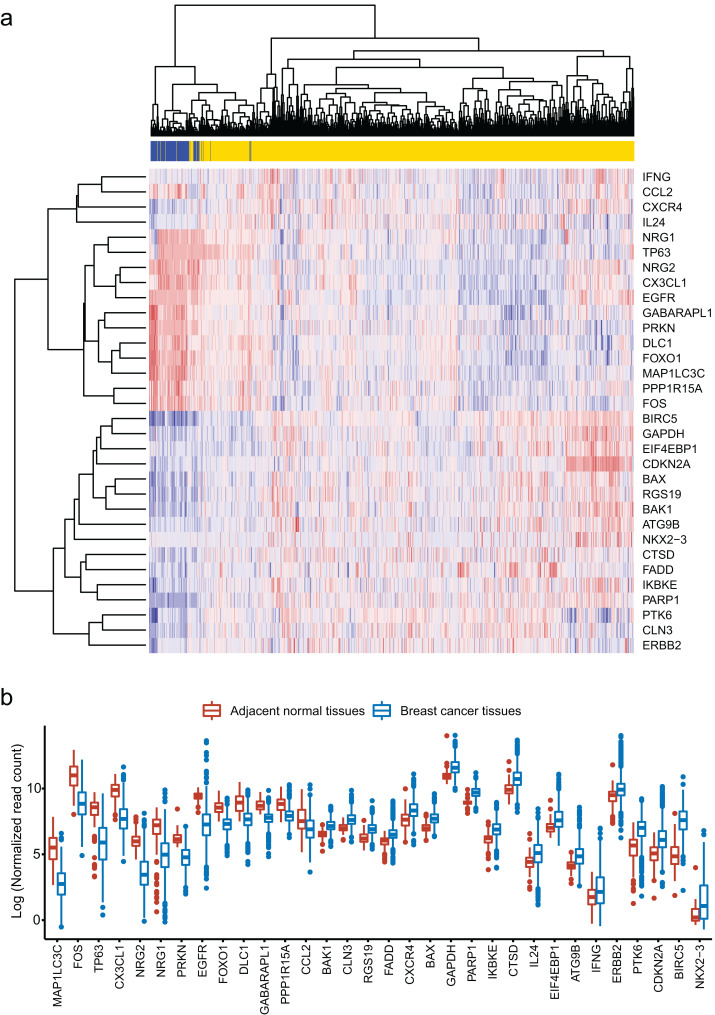
Identification of 32 differentially expressed autophagy-related genes (ARGs). (A) Heatmap of the 32 differentially expressed ARGs in breast cancer tissues compared to adjacent normal tissues. (B) Box plot of the 32 differentially expressed ARGs.

We obtained 232 ARGs from Human Autophagy Database. To obtain differentially expressed ARGs, we firstly compared 1,097 breast cancer tissues with 113 adjacent normal tissues and gained 6,168 differentially expressed genes. Then, we identified 32 differentially expressed ARGs from the 6,168 genes. The heatmap of these 32 ARGs is shown in Fig. 1A. Figure 1B shows the distribution of expression level of the 32 ARGs in breast cancer tissues and adjacent normal tissues.

### Functional annotation of the differentially expressed ARGs

**Figure 2:**
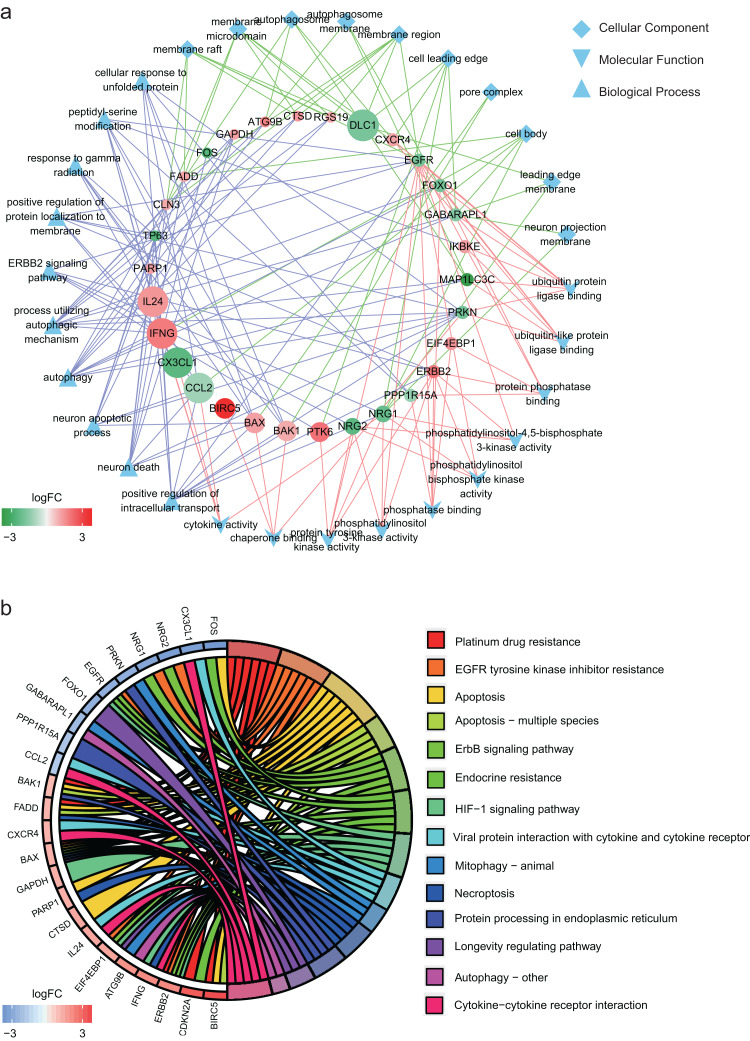
Functional annotation of the 32 differentially expressed autophagy-related genes (ARGs). (A) The top 10 gene ontology terms of each ontologies and the related ARGs. (B) KEGG enrichment analysis of the 32 differentially expressed ARGs.

In GO annotation, genes were annotated to three ontologies: cellular component, molecular function and biological process. The top 10 GO terms in each ontology and the related genes are shown in Fig. 2A. The three most commonly assigned GO terms for cellular component were membrane raft, membrane microdomain and autophagosome. The most commonly assigned GO terms in the molecular function were ubiquitin protein ligase binding, ubiquitin-like protein ligase binding and protein phosphatase binding. Positive regulation of intracellular transport, neuron death and neuron apoptotic process were the most commonly assigned terms for the biological process.

Figure 2B shows the result of KEGG enrichment analysis. The 32 ARGs are enriched 14 pathways and mainly enriched in platinum drug resistance, EGFR tyrosine kinase inhibitor resistance, apoptosis, apoptosis − multiple species and ErbB signaling pathway.

### Identification of a 6-ARG signature associated with prognosis

**Figure 3:**
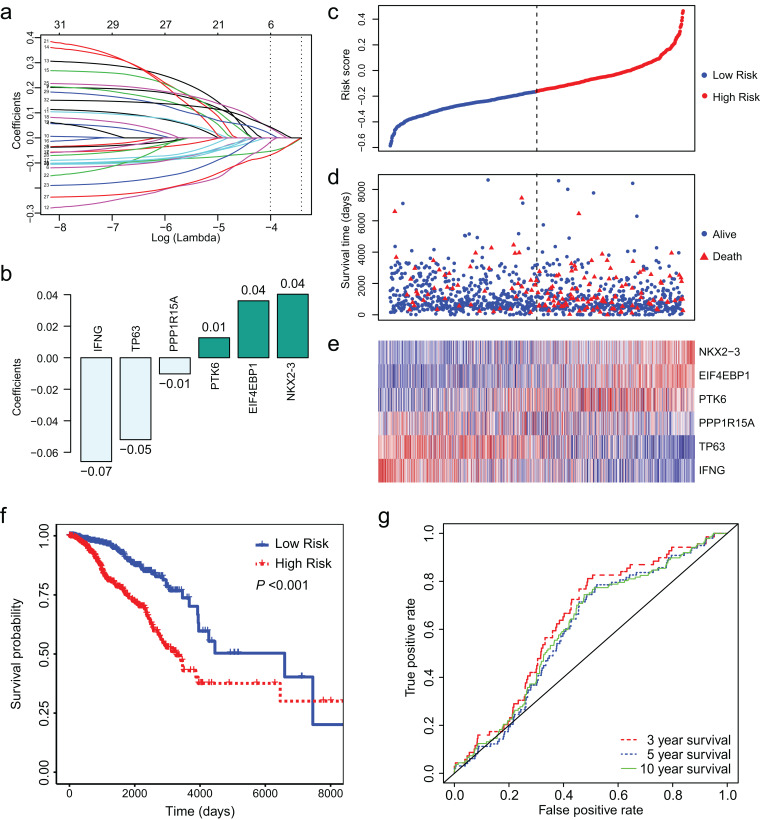
LASSO Cox regression of the 32 differentially expressed autophagy-related genes (ARGs). (A) LASSO coefficient profiles of the 32 differentially expressed ARGs. (B) Six ARGs and their coefficients. (C) The distribution of the risk score for the patients. (D) Survival time and status of the patients in the high-and low-risk groups, as defined by the risk score. (E) Expression patterns of the 6 ARGs. (F) The low-risk group has significantly longer survival times than high-risk group (HR = 2.56, 95% CI [1.81–3.61], *P* < 0.01). (G) Receiver operating characteristic (ROC) curve of the 6-ARG signature. The area under the ROC curve (AUC) was 0.64 for 3 year survival, 0.60 for 5 year survival, and 0.61 for 10 year survival. Dotted line in (C) and (D) represents the median of the risk score. The patients in (C), (D) and (E) were sorted by risk score in ascending order.

Least absolute shrinkage and selection operator regression was conducted to identify key ARGs from the 32 ARGs. Finally, we obtained 6 ARGs with non-zero coefficient (Fig. 3A). Figure 3B presents the 6 ARGs and their coefficients. We calculated risk score for each patient using the coefficients and (log2 (normalized read counts +1)). We separated the patients into low-and high-risk groups according to their risk score. Survival analysis showed that low risk score was associated with longer survival time and less deaths (Figs. 3C–3E). The median survival days was 6,593 for low-risk group and 3,126 for high-risk group (Fig. 3F, HR = 2.56, 95% CI [1.81–3.61], *P* < 0.01). We conducted ROC curve analysis to assess the prognostic performance of the 6-ARGs signature. The AUC was 0.64 for 3 year survival, 0.60 for 5 year survival, and 0.61 for 10 year survival (Fig. 3G).

### Validation of the 6-ARG signature in test dataset

**Figure 4:**
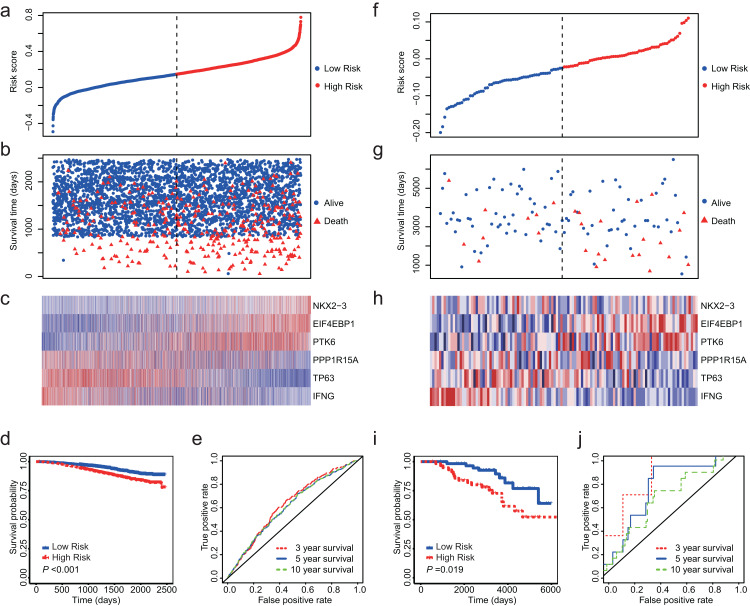
Validation of the 6-ARG signature in GSE96058 and GSE88770. (A) The distribution of the risk score in GSE96058. (B) Survival time and status of the patients in GSE96058. (C) Expression patterns of the 6 ARGs in GSE96058. (D) The low-risk group has significantly longer survival times than high-risk group in GSE96058 (HR = 1.83, 95% CI [1.47–2.27], *P* < 0.001) (E) Receiver operating characteristic (ROC) curve of the 6-ARG signature for GSE96058. The area under the ROC curve (AUC) was 0.63 for 3 year survival, 0.61 for 5 year survival, and 0.61 for 10 year survival. (F) The distribution of the risk score in GSE88770. (G) Survival time and status of the patients in GSE88770. (H) Expression patterns of the 6 ARGs in GSE88770. (I) The low-risk group has significantly longer survival times than high-risk group in GSE88770 (HR = 2.52, 95% CI [1.14–5.57], *P* = 0.02). (J) Receiver operating characteristic (ROC) curve of the 6-ARG signature for GSE88770. The area under the ROC curve (AUC) was 0.83 for 3 year survival, 0.72 for 5 year survival, and 0.64 for 10 year survival. Dotted line in (A), (B), (F) and (G) represents the median of the risk score. The patients in (A)–(C) and (F)–(H) were sorted by risk score in ascending order.

Totally, we obtained 10 datasets from GEO (Table 1). The outcome included disease-free survival (DFS), distant metastasis-free survival (DMFS), distant recurrence-free survival (DRFS), disease-specific survival (DSS) and OS. Since NKX2-3 is not detected by HG-U1334A, we were unable to calculate the risk core for the datasets produced by platform of HG-U1334A. GSE19783 did not detect TP63 and was also not used as test dataset. The observed outcomes of each dataset were listed in Table 1. Because GSE96058 and GSE88770 assessed OS, we firstly validated the 6-ARG signature in these two datasets. The both datasets showed that low risk score was associated with longer survival time and less deaths (Figs. 4A–4C and 4F–4H). Regarding GSE96058, patients with high risk score had a HR of 1.83 (95% CI [1.47–2.27], *P* < 0.01; Fig. 4D); The AUC was 0.63 for 3 year survival, 0.61 for 5 year survival, and 0.61 for 10 year survival (Fig. 4E). With respect to GSE88770, the HR of high-risk score patients is 2.52 (95% CI [1.14–5.57], *P* = 0.02; Fig. 4I) compared to low-risk score patients; The AUC was 0.83 for 3 year survival, 0.72 for 5 year survival, and 0.64 for 10 year survival (Fig. 4J).

**Table 1:** The datasets involved with gene expression data of breast cancer.

Dataset	Platform	Subjects	Included patients	Outcome
GSE96058	Illumina HiSeq 2000 Illumina NextSeq 500	3,409 Breast cancer patients	3,409	OS
GSE88770	HG-U1334 plus 2	117 Patients with invasive lobular carcinoma	117	OS, DRFS
GSE19615	HG-U1334 plus 2	115 Breast cancer patients	115	DRFS
GSE21653	HG-U1334 plus 2	266 Early breast cancer patients	248	DFS
GSE2990	HG-U1334A	189 Invasive breast carcinomas	187 (RFS), 179 (DMFS )	DFS, DMFS
GES3494	HG-U1334A	251 Breast cancer patients	236	DSS
GSE7390	HG-U1334A	198 Lymph node-negative breast cancer patients without systemically treatment	198	OS, DFS, DMFS
GSE17705	HG-U1334A	298 ER-positive patients treated with tamoxifen for 5 years	298	DFS
GSE25066	HG-U1334A	508 HER2-negative breast cancer cases treated with taxane-anthracycline chemotherapy pre-operatively and endocrine therapy if ER-positive	507	DRFS
GSE19783	Agilent-014850	115 Breast cancer patients	112	DFS

**Note:**

OS, overall survival; DRFS, distant recurrence-free survival; DFS, disease-free survival; DMFS, distant metastasis-free survival; DSS, disease-specific survival.

We also validated the 6-ARG signature in other outcomes. After analyzing GSE21653, we found that low-risk score patients had a better DFS than high-risk score patients (HR = 2.36, 95% CI [1.48–3.76], *P* < 0.01). However, we did not find an association between the 6-ARG signature and DRFS (HR = 1.39, 95% CI [0.48–4.01], *P* = 0.54 for GSE19615; and HR = 1.56, 95% CI [0.72–3.40], *P* = 0.26 for GSE88770).

### ARGs associated with breast cancer survival

We identified 170 ARGs associated with breast cancer survival (Table S1). Among these genes, 18 ARGs had significant associations in 5 or more calculations. The top 3 ARGs with highest numbers of significant associations were EIF4EBP1, FOS and FAS. EIF4EBP1 showed a significant harmful effect in eight calculations. FAS and FOS showed significant beneficial effects in seven calculations.

### Expression of ARGs in different molecular subtypes and grade of breast cancer

**Figure 5:**
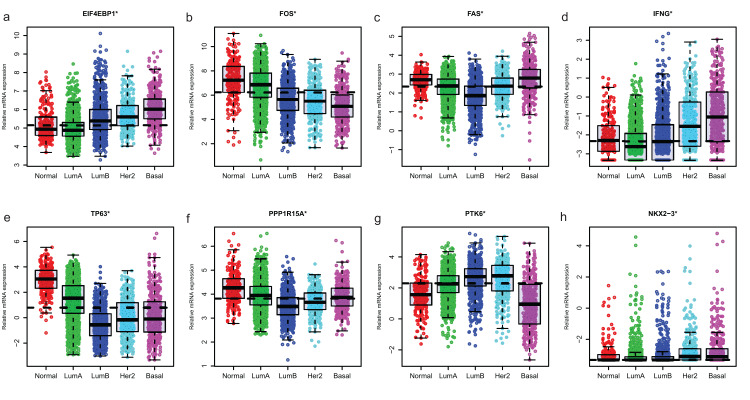
The expression levels of eight autophagy-related genes in different molecular subtypes of breast cancer in GSE96058. (A) EIF4EBP1. (B) FOS. (C) FAS. (D) IFNG. (E) TP63. (F) PPP1R15A (G) PTK6. (H) NKX2−3. *P < 0.05.

**Figure 6:**
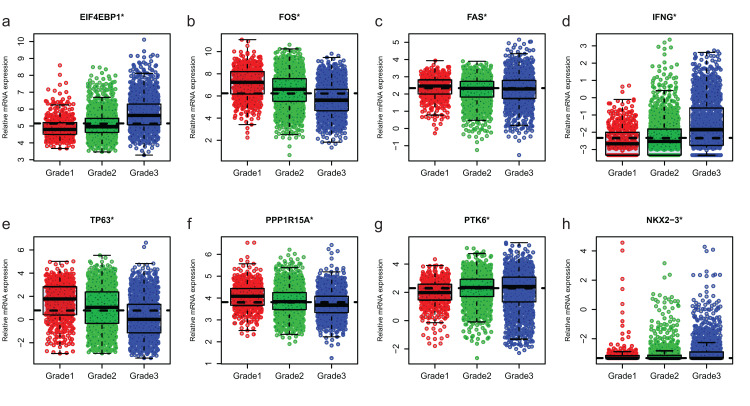
The expression levels of eight autophagy-related genes in different Nottingham histologic grade of breast cancer in GSE96058. (A) EIF4EBP1. (B) FOS. (C) FAS. (D) IFNG. (E) TP63. (F) PPP1R15A (G) PTK6. (H) NKX2−3. *P < 0.05.

Since GSE96058 had detailed clinical information and largest sample size, we explored the expression of the 8 ARGs including those in the 6-ARG signature and the top 3 ARGs in Table S1. When analyzed according to molecular subtypes (Normal, LumA, LumB, Her2 and Basal), all the 8 ARGs showed a significant correlation with molecular subtypes. As the malignancy of breast cancer increased, the expression of several ARGs, such as EIF4EBP1, FOS and TP63, decreased or increased (Fig. 5). The significant correlations were also found between Nottingham histologic grade and expression levels of the 8 ARGs. The expression level of several ARGs also showed a trend with grade from grade 2 to grade 4 (Fig. 6).

## Discussion

Autophagy has been reported to either inhibit or promote cancer cell proliferation or tumorigenesis. In most case, cancers upregulate autophagy to survive microenvironmental stress and to increase growth and aggressiveness (White, 2015). In present study, we identified 32 differentially expressed ARGs after comparison of 1,097 breast cancer tissues and 113 adjacent normal tissues based on TCGA. The GO terms and pathways annotated in enrichment analyses may help us to understand the function of these 32 differentially expressed ARGs. We constructed a 6-ARG signature predicting breast cancer survival using LASSO regression. Then, the 6-ARG signature was validated using datasets from GEO, and the results showed that 6-ARG signature can predicate OS and DFS of breast cancer patients but not DRFS. There may be several reasons attributed to this result. Firstly, 6-ARG signature was indeed only associated with OS and DFS but not DRFS. Secondly, the relative small sample size of GSE19615 and GSE88770 explain the lack of a statistical significance for DRFS. Thirdly, different population and different subtype and stage of breast cancer may be another reason. Taken together, 6-ARG signature can be used to predicate survival of breast cancer patients.

Studies have showed that the six genes in the signature involved in every aspect of cancer. Interferon gamma (IFNG) has been known to regulate tumor immune surveillance and tumorgenesis (Dong et al., 2015). Yaghoobi et al. (2018) showed that IFNG had a slightly higher expression level in breast cancer tissues compared with their adjacent non-cancerous tissues, but IFNG expression was significantly higher in grade 1 samples compared with grade 2 ones. Tumor protein 63 (TP63), known as a member of the p53 family, maintains stem cell pluripotency and suppresses the metastatic potential of cancer cells by regulating genomic programs (Abbas et al., 2018). High levels of TP63 was associated with increased metastasis-free survival of breast cancer patients (Papageorgis et al., 2015). Protein phosphatase 1 regulatory subunit 15A (PPP1R15A), also known as GADD34, is a stress-inducible gene and promotes cell death following proteasome inhibition via enhancing protein synthesis to activate death-associated mechanisms (Liu et al., 2015). Protein tyrosine kinase 6 (PTK6) is a non-receptor tyrosine kinase and its downregulation induces apoptosis of Lapatinib-resistant Her2+ breast cancer cells (Park et al., 2015). Increased PTK6 expression promoted metastasis and progression of breast cancer (Ito et al., 2016; Regan Anderson et al., 2013). PTK6 also regulates growth and survival of endocrine therapy-resistant ER+ breast cancer cells (Ito et al., 2017). NK2 homeobox 3 (NKX2-3) encodes a member of the NKX family of homeodomain transcription factors. A study reported that Nkx2-3 contributed to the pathogenesis of colorectal cancer by regulating the Wnt signaling pathway (Yu et al., 2010). EIF4EBP1 (also known as 4EBP1) acts as a crucial effector in mTOR signaling pathway and has been shown involved in cancer development and progression (Cha et al., 2015). Karlsson et al. (2013) showed that EIF4EBP1 are correlated and significantly related to poor outcome in four independent breast cancer cohorts. These results support our finding. Further studies are needed to confirm the roles of these genes in breast cancer.

We obtained 170 ARGs associated with survival of breast cancer patients. Several ARGs have been shown involved in pathogenesis of breast cancer. ATG9B has lower expression level in invasive breast tumors than matched normal tissues (Claude-Taupin et al., 2018; Zhang et al., 2016). ATG9A inhibition led to an inhibition of in vitro cancer features (Claude-Taupin et al., 2018). GABARAPL1, as a tumor suppressor protein in breast cancer, is associated with a better outcome for patients with lymph node-positive breast cancer (Berthier et al., 2010; Poillet-Perez et al., 2017). An inverse relationship was also observed between GABARAPL1 mRNA and tumor stage (Hervouet et al., 2015). Among the 170 ARGs, EIF4EBP1, FOS and FAS were the top 3 ARGs with highest numbers of significant associations. EIF4EBP1 had a higher expression level in patients with more malignant molecular subtypes and higher grade breast cancer. Combined the finding in constructing ARG signature, EIF4EBP1 may be a key ARG for breast cancer.

The potential limitations of the present study need to be considered when interpreting the results. Firstly, raw read count of GSE96058 was not provided, therefore the expression profile may be processed in a different method from our analysis. In addition, the signal generated by microarray is different from RNA-seq. As a result, we were unable to used same cutoff to separate patients into low- and high-risk groups. Secondly, as several datasets did not detect expression of all the six genes in the signature, we were unable to calculate the risk score for these datasets. Further studies are warranted to validate the findings of this study.

## Conclusion

In conclusion, our 6-ARG signature was of significance in predicting of overall survival of patients with breast cancer. The 6-ARG signature may help to distinguish poor prognosis patients from patients with long-term survival. Further analyses are needed to investigate the molecular mechanisms by which the six genes affect survival of breast cancer patients.

## Supplemental Information

10.7717/peerj.9621/supp-1 Supplemental Information 1 The genes associated with survival of breast cancer patients.

## Additional Information and Declarations

### Data Availability

The following information was supplied regarding data availability: All the data used in the present study are available at TCGA (search term: BRCA) and GEO: GSE96058, GSE88770, GSE19615, GSE21653, GSE2990, GES3494, GSE7390, GSE17705, GSE25066, GSE19783.
